# Curiosities of the Vegetable Kingdom

**Published:** 1891-09

**Authors:** 


### CURIOSITIES OF THE VEGETABLE KINGDOM.

A species of vegetable intoxicant, writes a correspondent, has been added to the collection of plant curiosities at the Washington Botanical Garden. The liquor it distills in the pitcher-shaped receptacles that hang from its stems is especially liked by frogs, which hop into these traps for the purpose of drinking it. Although the sweetish fluid is a powerful intoxicant, the batrachian customer, however wildly over stimulated, would certainly jump out again were it not that two very sharp dagger-like thorns project downward from the lip of the vessel in such a manner that Mr. Frog, in trying to escape, is thrust through the body by them at every leap, until presently he falls dead in the "liquid refreshment"—an appropriate object lesson to all intemperate creatures—whereupon the plant absorbs his substance, as the ordinary whiskey shop consumes that of its frequenter, and is thus supported.

This species is tropical, and has to be kept in the greenhouse devoted to plants of the equatorial belt. Naturally there are no frogs in the conservatory, and so Superintendent Smith is obliged to feed this eccentric vegetable with raw meat, chopped fine, on which it thrives excellently. For lack of insects, likewise he supplies with the same artificial sustenance, the other sorts of dram shops that find in bugs of various descriptions their chosen prey. They will all eat beef, although each variety seems to have in nature its particular line of customers, one capturing cockroaches, another ants, and so on. Doubtless they could all live on any animal food, but there seems to be a difference of taste among insects as to the liquors. One species will only touch the drink served by a certain representative of this carnivorous plant family; another selects, by preference, a different brew, and so on. Thus but one sort of bug is ordinarily found in each set of pitchers, those designed for the accommodation of large beetles and cockroaches being as big as small shoes. The structure of each trap is, beyond measure, curious, the inner surface of some being coated with little bristles that project downward, and prevent the guest so hospitably received from walking out again. This is particularly a feature of what the superintendent calls the vegetable lager beer saloons, which prepare a liquor of much less intoxicating quality in tall chalice-shaped vessels instead of pitchers, depending upon drowning their customers rather than upon making them so drunk that they cannot get away.

So far as can be ascertained, no analysis has ever been made of the liquors dispensed by them, chiefly owing to the fact that such an experiment would involve a very difficult problem in organic chemistry. It is known, however, that the strongest of them contain a large proportion of alcohol.

A novelty at the Botanic Gardens is a plant whose leaf bears a remarkably well executed caricature of the Duke of Wellington, all done in the veining ; but in the interest of visitors it does not seriously rival either the "mother-in-law plant," a scrap of which swells up your tongue so that you cannot speak for days, or the famous "butcher plant," of Maryland, that has, instead of leaves, so many pairs of toothed jaws that close upon any insect venturing between to get at the bait within.

This "butcher plant," which grows nowhere in the world save in the vicinity of Wilmington, N. C., suffers for its carnivorous habits by being a chronic victim of indigestion.

Each stomach trap, having used up most of the gastric juice which it secretes in digesting the first living prey caught, usually finds the second victim it captures disagree with it, and the third it is unable to assimilate satisfactorily. Then the trap turns from green to brown and dies, like any leaf, other fresh ones developing meanwhile to take up the work of gobbling. After all, this greedy vegetable is not nearly so bad as the "cruel plant," as it is called, whose flowers wantonly capture unsuspecting butterflies that alight to sip honey, and hold them until they are dead, when the grasp of the ruthless petals is relinquished, and the luckless visitor is dropped on the ground.

Some plants employ insects as their servants in the work of reproducing their species, paying them wages in honey. Most vegetables combine the two sexes in one flower ; but breeding "in and in" is no more healthy for them than it is for animals. One blossom must marry with another if the species is to be continued in a healthy way. So the young honeysuckle dresses himself in a Spring suit of bright yellow, and perfumes himself deliciously for the purpose of attracting the gay butterflies that flutter around. He also provides a small store of nectar in a golden cup to offer any insect guest that may come his way. Presently a butterfly pauses to take a sip of the sweet liquor, but in doing so she cannot avoid getting some of the pollen on her head, and this she carries to another honeysuckle, where she stops for a second bit of refreshment, and incidently rubs off some of the pollen upon its stigma. Thus is accomplished the marriage of the flowers.

But the bee is the Cupid of the vegetable world, to whom is assigned most of this marrying and giving in marriage among the blossoms. There is one kind of orchid that depends altogether for the continuance of its species upon flights among bees. To a moral delinquency on their part it may be said to owe its survival entirely. The petals of each of its flowers are so bent as to form a sort of little tunnel, and to get at the honey a bee must go in at one end or the other. If nothing interferes it will never come in contact with any of the pollen, but now and then it happens that it meets another bee which has entered from the other side. Then there is a fight, and in the scrimmage the combatants get bounced around, and are covered with the reproductive powder. However, in order to accomplish any thing, one of these bees must go off and have the same sort of fight in another orchid blossom, so as to transfer a portion of the pollen to the stigma. Luckily this occurs often enough to perpetuate the plant.

Some kinds of orchids imitate to the life bees, butterflies and moths, apparently for the purpose of attracting these insects on the decoy duck principle The object is not quite so evident in the case of varieties of these extraordinary plants whose flowers counterfeit with amazing exactness toads, huge spiders, and other animals. There is one which presents the likeness of a man hanging by the head, and another that opens and shows a beautiful dove in an enclosure of petals.

A book might be made of the freak plants of the world. There is the vegetable boa constrictor of India, known as the "maloo climber," which twines about great trees and strangles them to death, so that they decay, fall in, and often leave the empty tower of climbers standing erect. In South America there is a "cow tree," which gives milk that is shown by chemical analysis to be of almost exactly the same composition as that of the cow, which it resembles to perfection in appearance and quality, tasting like sweet cream. On the elevated barren plains west of the Volga grows a plant closely resembling a lamb, which was said by travellers of old to bend upon its stalk to feed upon the herbage about it.

